# High‐throughput sequencing outperforms traditional morphological methods in Blue Catfish diet analysis and reveals novel insights into diet ecology

**DOI:** 10.1002/ece3.7460

**Published:** 2021-03-30

**Authors:** Heather K. Evans, Aaron J. Bunch, Joseph D. Schmitt, Frederick J. Hoogakker, Kara B. Carlson

**Affiliations:** ^1^ Genomics and Microbiology Laboratory North Carolina Museum of Natural Sciences Raleigh NC USA; ^2^ North Carolina Wildlife Resources Commission Raleigh NC USA; ^3^ Virginia Department of Wildlife Resources Charles City VA USA; ^4^ Department of Forestry and Environmental Conservation Clemson University Clemson SC USA; ^5^ Virginia Polytechnic Institute and State University Blacksburg VA USA; ^6^ U.S. Geological Survey Great Lakes Science Center Sandusky OH USA; ^7^ Tennessee Cooperative Fishery Research Unit Tennessee Tech University Cookeville TN USA; ^8^ Department of Genetics North Carolina State University Raleigh NC USA

**Keywords:** at‐risk species, Blue Catfish, diet, flow rate, high‐throughput sequencing, metabarcoding

## Abstract

Blue Catfish *Ictalurus furcatus* are an invasive, yet economically important species in the Chesapeake Bay. However, their impact on the trophic ecology of this system is not well understood. In order to provide in‐depth analysis of predation by Blue Catfish, we identified prey items using high‐throughput DNA sequencing (HTS) of entire gastrointestinal tracts from 134 samples using two genetic markers, mitochondrial cytochrome c oxidase I (COI) and the nuclear 18S ribosomal RNA gene. We compared our HTS results to a more traditional “hybrid” approach that coupled morphological identification with DNA barcoding. The hybrid study was conducted on additional Blue Catfish samples (*n* = 617 stomachs) collected from the same location and season in the previous year. Taxonomic representation with HTS vastly surpassed that achieved with the hybrid methodology in Blue Catfish. Significantly, our HTS study identified several instances of at‐risk and invasive species consumption not identified using the hybrid method, supporting the hypothesis that previous studies using morphological methods may greatly underestimate consumption of critical species. Finally, we report the novel finding that Blue Catfish diet diversity inversely correlates to daily flow rates, perhaps due to higher mobility and prey‐seeking behaviors exhibited during lower flow.

## Graphical abstract

We compared high‐throughput sequencing (HTS) to traditional morphological diet analysis methods in Blue Catfish and found that HTS vastly outperforms visual methods, even when coupled with barcoding of unidentified tissues. We identified levels of at‐risk species consumption, including Atlantic Sturgeon and alosines, by Blue Catfish higher than those previously reported. Additionally, our study details the novel finding that Blue Catfish diet diversity is inversely related to flow rates.

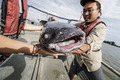

## 1 INTRODUCTION

**FIGURE 1:**
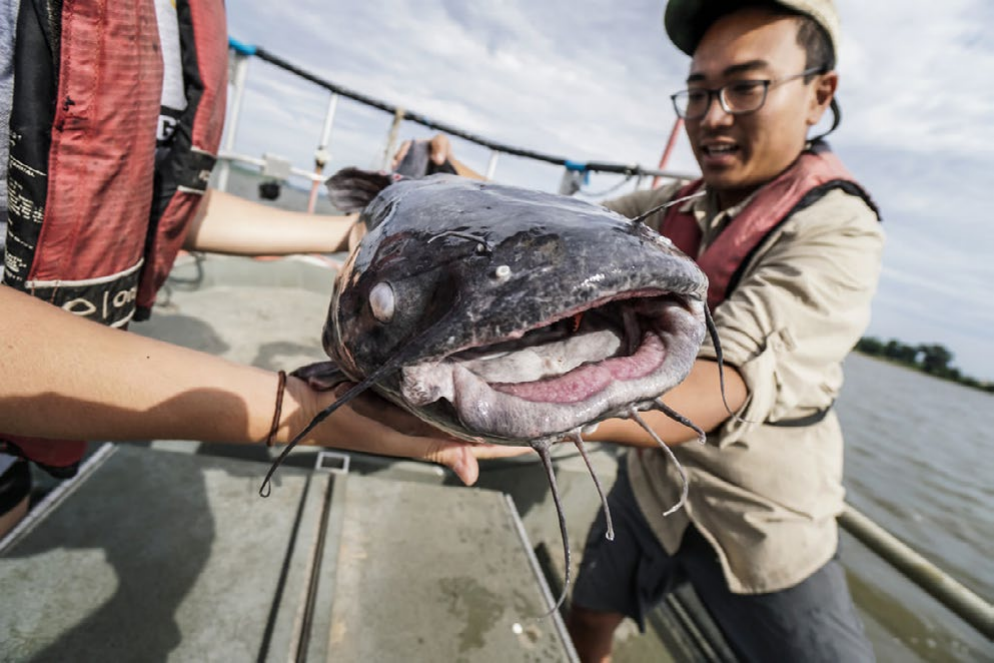
Blue Catfish *Ictalurus furcatus* collected from the Chesapeake Bay by Joseph Schmitt and Hae Kim

Blue Catfish *Ictalurus furcatus* (Figure 1) are an invasive species found in high abundance throughout the Chesapeake Bay (Bunch et al., 2018; Fabrizio et al., 2018). Originally introduced to establish a fishery, Blue Catfish expanded throughout the Chesapeake Bay ecosystem (Schloesser et al., 2011). Blue Catfish have been documented in excess of 5 feet and 100 pounds (Graham, 1999). Size combined with the relative abundance of this omnivorous species presents a concern for management agencies as Blue Catfish are known to prey on native species such as economically important Blue Crab *Callinectes sapidus* and threatened alosines (Iwanowicz et al., 2019; Schmitt et al., 2017). Additionally, Blue Catfish can outcompete native species for resources, and White Catfish *Ameiurus catus* populations have declined as Blue Catfish populations have increased (Chesapeake Bay Program, 2020).

Recent studies have sought to address the full impact of Blue Catfish feeding ecology in its non‐native range using visual, morphometric methods (Waters et al., 2004), sometimes in combination with DNA barcoding of specific tissues (Aguilar et al., 2017; Moran et al., 2015; Schmitt et al., 2017). Such studies are difficult to use in quantifying the effect of Blue Catfish predation on ecosystems as taxonomic identification of prey items is highly dependent upon tissue degradation, thus limiting prey identification to those items only recently consumed and of sufficient mass to withstand rapid digestive processes (Rees et al., 2020; Su et al., 2018). Studies indicate that soft‐tissue prey, including larval and juvenile fishes, are unrecognizably digested within a span of twenty minutes to a few hours of consumption (Bromley, 1994; Carreon‐Martinez et al., 2011).

The use of high‐throughput sequencing (HTS) for diet studies is becoming more widely accepted (Casey et al., 2019; Rees et al., 2020; Waraniak et al., 2019). Such methods perform better than morphological observation, especially when diet items, including at‐risk or economically important species, are partially or fully digested with no assemblance of an organism for viewing, as mentioned above (Schwarz et al., 2018; Tverin et al., 2019; Waraniak et al., 2018). Researchers can describe diverse prey assemblages (Bessey et al., 2019; Sousa et al., 2016), analyze non‐native species’ diets in newly invaded habitats (Harms‐Tuohy et al., 2016), and quantify trophic interactions (Casey et al., 2019) using HTS methods. Despite the benefits of HTS to morphological studies and the importance of Blue Catfish from both a conservation and economic viewpoint, only one study using HTS to analyze the diet of Blue Catfish in a limited numbers of samples (*n* = 12) has been published to date (Iwanowicz et al., 2019).

In this study, we sought to gain a holistic view of diet behavior and of the potential impact of invasive Blue Catfish predation in a Chesapeake Bay tidal river ecosystem using high‐throughput sequencing methods. To accomplish this objective, we conducted HTS of 134 Blue Catfish representing multiple life stages that were collected in the fall of 2016 from the Pamunkey River, Virginia. High‐throughput sequencing was employed on material collected from the whole gut, stomach to anus, using 18S and COI, genes commonly employed in diet analyses (Leray & Knowlton, 2015; Zhan et al., 2014). We examined the effect of marker choice on taxonomic identification of prey items and investigated diversity metrics in relation to individual and environmental covariates. Additionally, we sought to quantitatively compare our results using HTS methods to a Blue Catfish diet study that employed a hybrid approach using visual observations of stomach content coupled with barcoding of single, yet unidentifiable tissues (Schmitt et al., 2018).

## 2 METHODS

### 2.1 Field collections

In fall 2016 (September–October), we obtained 136 Blue Catfish samples ranging in size from 170 mm to 770 mm TL from two sampling sites located three km apart (hereafter referred to as Chericoke Retreat and Putney's Mill) on the Pamunkey River, VA. All sampling occurred in tidal‐fresh habitats with minimal salinity influence (0–0.1 ppm) at 65 rkm (measured from the York River mouth at the Chesapeake Bay). Each collection transect was approximately 2 km each in length. We used a boat‐mounted Smith‐Root electrofishing 7.5 GPP unit, 5,000‐watt generator at 15 pulses per second. All fish were measured (TL, in mm) and placed into containers with ice to minimize tissue degradation. On the same date, fish were taken to a controlled laboratory setting for GI tract extraction and preservation. Entire GI tracts (esophagus to anus) were carefully removed with sterile procedures, placed in 100% ethanol, and stored in a temperature‐controlled facility until further processing.

### 2.2 Genomic DNA extraction and library preparation

We briefly examined contents from the GI tract and removed them using sterile techniques. Two Blue Catfish contained empty stomachs and digestive tracts and were not included in further analysis. DNA was extracted using the Macherey Nagel Stool Sample Extraction kit. Amplification of barcoding regions was conducted for COI (Leray et al., 2013) and for 18S (Zhan et al., 2014). We employed a species blocking primer 5′‐CAAGAATCAGAAAAGGTGTTGGTAAAGA‐3′ as outlined in Leray et al. (2013) for COI to limit amplification of the host DNA. Illumina adaptors were added to PCR products to uniquely identify each sample and barcoding gene. After cleaning, products were sequenced using an Illumina MiSeq Reagent Kit v3 (600‐cycle).

### 2.3 High‐throughput DNA sequencing (HTS) analysis

Sequence reads obtained from the MiSeq platform were analyzed using QIIME 2‐2019.4 (Bolyen et al., 2019), with 18S and COI sequences split into two separate groups for processing. For each run, primer sequences were trimmed and forward and reverse reads joined and filtered using DADA2 (Callahan et al., 2016). Singletons were removed, and any reads identified in the negative control were subtracted from other samples. Sequences from all six runs were then combined into one table and clustered at 98% using Vsearch (Rognes et al., 2016). Taxonomy was assigned by comparing 18S features against the SILVA 132 database (695,171 sequences, Quast et al., 2013) in QIIME 2, while COI taxonomy was assigned using BLAST + local alignment (Camacho et al., 2009) with a reference database containing 1,769,786 COI sequences downloaded from NCBI. One feature representing the host species was removed from each sample to eliminate host‐introduced bias in the diet analysis.

### 2.4 Diet diversity and composition

We produced alpha‐rarefaction curves for 18S and COI using QIIME 2. For 18S reads, samples were rarefied to 1,355 while COI reads were rarefied to 5,403. Alpha‐ and beta‐diversity analyses were run using a rooted tree in QIIME 2 on rarefied tables. Kruskal–Wallis tests of alpha diversity matrices were performed, and q values (*p* values with a Benjamini–Hochberg correction, Benjamini & Hochberg, 1995) of less than 0.05 were accepted as statistically significant. For diversity metrics analyzing size, fish were grouped by 100 mm increments. Beta‐diversity comparisons were run with PERMANOVA using a Bray–Curtis and/or a weighted unifrac distance matrix and Adonis as implemented in QIIME 2. Differential abundance was analyzed with ANCOM (Mandal et al., 2015) on nonrarefied tables. Taxonomy bar plots were created using nonrarefied, collapsed taxonomy tables.

We compared the results from our HTS molecular approach to data obtained from a previous hybrid study conducted by Schmitt et al. (2018). This hybrid dataset comprised 617 Blue Catfish diet samples collected in the same general location as our study sites on the Pamunkey River during the months of September and October in 2015. Richness, diversity, and diet composition were compared to HTS results across sampling dates and life stages. Life stage was split by juvenile (up to 300 mm) and adult (over 300 mm). Plots were constructed using the “ggplot2” package in R (Wickham, 2016).

We correlated diet diversity metrics to daily flow measurements (USGS 01673000 gaging station near Hanover, VA). The hybrid study analyzed contents found in stomachs only, which would have been consumed within the past 24 hr. Therefore, we correlated the hybrid dataset to average flow within one day of collections. (Carreon‐Martinez et al., 2011). We estimated average flow rate for HTS analysis using the previous three days of each sampling event. We based this decision on a study by Schultz et al. (2015) that demonstrated gut evacuation rate in salmonids was approximately 2 days at 22°C and decreased as temperatures decreased. As the average temperature over our sampling dates was 19.4°C, we felt three days were a more accurate predictor for persistence of diet items within the gut for our samples.

Cumulative prey curves for both genes and approaches were constructed to identify the number of samples needed to characterize Blue Catfish diets. Curves and associated 95% confidence intervals were calculated with EstimateS, version 9.1 (Colwell, 2013), where the cumulative number of unique taxa were plotted against the randomly pooled samples. This process was bootstrapped 1,000 times to generate means and 95% confidence intervals. We used the mean slope (B) of the last five subsamples (linear regression) as an objective criterion for sample size sufficiency, where sample size is considered sufficient when B ≤ 0.05 (Bizzarro et al., 2007; Brown et al., 2012).

## 3 RESULTS

### 3.1 Taxonomic assessment

After denoising and clustering at 98%, we detected 1,630 18S features with a mean frequency per sample of 7,494. An average of 35.4 features (22.7 unique taxa, hereafter referred to as operational taxonomic units, OTUs) was detected per sample. We identified 8,701 COI features with a mean frequency per sample of 30,269. An average of 141.2 features (45.3 OTUs) was identified per sample. The COI gene thus detected, on average, two times more OTUs than the 18S gene in Blue Catfish.

### 3.2 Blue catfish diet analysis

**FIGURE 2:**
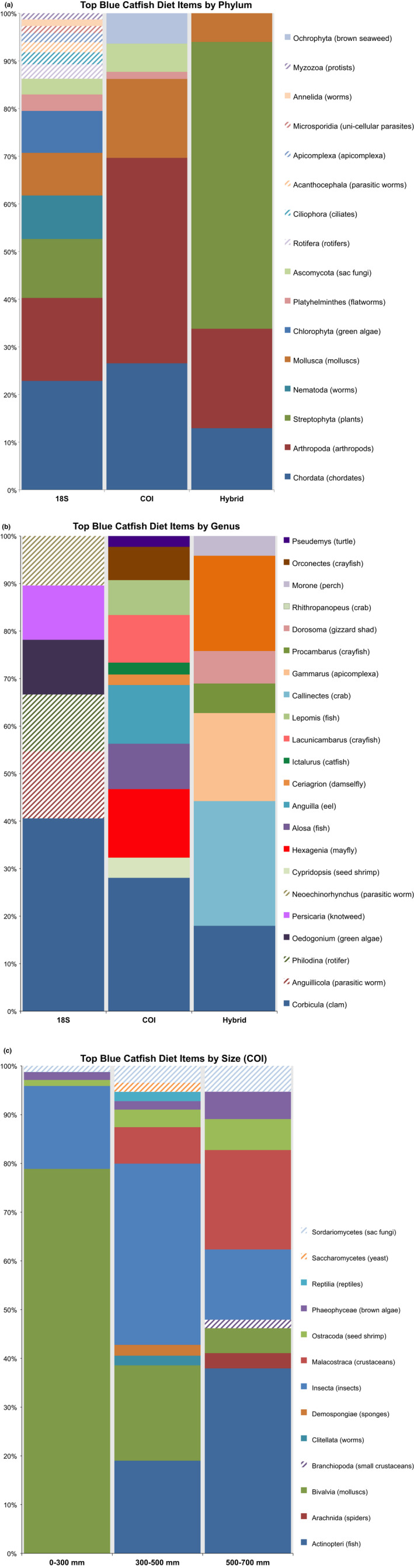
Blue Catfish diet taxonomic bar plots. Any assigned diet items constituting at least one percent of total diet are shown. Bar plots have been scaled to illustrate the relative proportions of each of these top diet items. Comparison of diet content at phylum (a) and genus (b) levels using either COI, 18S, or the hybrid method illustrates how examined diet content varies depending on methodology. Additionally, COI sequences revealed two ontogenetic shifts in our dataset (c). As Blue Catfish transition from juveniles to adults, we observed a decrease in consumption of Asian Clam. At 500 mm, Blue Catfish began to shift toward piscivory as they decreased plant and mollusc consumption and increased fish and crayfish predation

Blue Catfish prey assessment revealed a highly diverse diet that included plant matter, fish, crayfish, turtles, terrestrial insects, aquatic macroinvertebrates, molluscs, algae, and several microscopic phyla (Figure 2). According to Faith's phylogenetics diversity (PD), fish collected on September 23 or October 20 had more diverse diets than those collected on October 3 or October 12. Faith's PD did not differ between collection sites, size of adult fish, or life stage of fish (juveniles versus adults). Weighted unifrac pairwise tests for both 18S and COI detected differences in diet for the sampling date groupings mentioned above (Table 1). COI sequences also detected distinct diets for collection sites (pseudo‐*F* = 2.64, q = 0.008) as well as for life stage (pseudo‐*F* = 5.30, q = 0.001), with juvenile Blue Catfish consuming noticeably more Asian Clam than their adult counterparts (Figure 2c). Additionally, COI sequences indicate a second diet shift for adults at around 500 mm TL (Table 2) as Blue Catfish began to decrease mollusc and plant intake and increase their consumption of fish as well as crayfish (Figure 2c). No differences were detected using 18S weighted unifrac matrices for collection site, size, or life stage. PERMANOVA (Adonis) analyses indicate that approximately 9.5% of variation can be attributed to the single variable of collection date (Table 3). Collection date in conjunction with collection site could explain an additional 3.9% to 4.5% of the variation observed. COI sequences indicated 3.7% of variation could be attributed to life stage and 6.1% to size of fish.

**TABLE 1:** Blue Catfish beta‐diversity. Pairwise PERMANOVA tests were run using weighted unifrac distance matrices for 18S and COI sequences. Differences in diet were detected with both genes between the two dates with the lowest flow rates versus the two dates with highest flow rates. COI sequences additionally indicated diet variation between life stage, collection site, and size of fish. N1 = sample size for Group1; N2 = sample size for Group2

	18S						COI					
	Group 1	N1	Group 2	N2	pseudo‐*F*	q‐value	Group 1	N1	Group 2	N2	pseudo‐ *F*	q‐value
Collection date	9/23/16	10	10/3/16	25	2.825	0.040	9/23/16	12	10/3/16	27	4.246	0.002
	9/23/16	10	10/12/16	22	2.689	0.041	9/23/16	12	10/12/16	37	5.305	0.002
	9/23/16	10	10/20/16	32	1.424	0.176	9/23/16	12	10/20/16	35	3.870	0.002
	10/3/16	25	10/12/16	22	0.879	0.435	10/3/16	27	10/12/16	27	1.663	0.104
	10/3/16	25	10/20/16	32	4.722	0.003	10/3/16	27	10/20/16	37	3.870	0.002
	10/12/16	22	10/20/16	32	5.043	0.003	10/12/16	37	10/20/16	35	3.620	0.005
Life stage	Adult	80	Juvenile	9	1.267	0.258	Adult	101	Juvenile	10	5.303	0.001
Collection site	Chericoke Retreat	35	Pamunkey's Mill	54	1.576	0.101	Chericoke Retreat	50	Pamunkey's Mill	61	2.636	0.008
Adult total length	300–399 mm	13	400–499 mm	23	1.107	0.477	300–399 mm	17	400–499 mm	31	1.953	0.135
	300–399 mm	13	500–599 mm	31	0.591	0.863	300–399 mm	17	500–599 mm	38	2.621	0.021
	300–399 mm	13	600–699 mm	12	1.266	0.464	300–399 mm	17	600–699 mm	14	2.659	0.029
	400–499 mm	23	500–599 mm	31	0.661	0.823	400–499 mm	31	600–699 mm	38	0.841	0.562
	400–499 mm	23	600–699 mm	12	1.141	0.477	500–599 mm	31	600–699 mm	14	1.393	0.255
	500–599 mm	31	600–699 mm	12	0.986	0.534	300–399 mm	38	500–599 mm	14	1.428	0.255

**TABLE 2:** Species of interest observed in Blue Catfish diets. At‐risk, indicator, and invasive species for the Chesapeake Bay ecosystem are noted here, along with the number and percentage [*n* (%)] of stomachs in which said species were identified. A comparison of these observations using high‐throughput sequencing versus hybrid methodology highlights the extent to which such species of critical interest may be underestimated using traditional morphological techniques for diet analysis

Common, Scientific name	Type	Species designation	Blue Catfish (HTS)	Blue Catfish (Hybrid)
American Eel, *Anguilla rostrata*	Fish	At‐risk	21 (15.4)	0 (0.0)
American Shad, *Alosa sapidissima*	Fish	Indicator, At‐Risk	10 (7.4)	0 (0.0)
Asian Clam, *Corbicula fluminea*	Bivalve	Invasive	81 (59.6)	15 (3.8)
Bay Anchovy, *Anchoa mitchilli*	Fish	Indicator	25 (18.4)	0 (0.0)
Blue Catfish, *Ictalurus furcatus*	Fish	Invasive	42 (30.9)	2 (0.1)
Blue Crab, *Callinectes sapidus*	Crustacean	Indicator	1 (0.7)	36 (0.1)
Flathead Catfish, *Pylodictis olivaris*	Fish	Invasive	1 (0.7)	0 (0.0)
Hydrilla, *Hydrilla verticillata*	Plant	Indicator, Invasive	17 (12.5)	0 (0.0)
Red Swamp Crayfish, *Procambarus clarkii*	Crustacean	Invasive	8 (5.9)	0 (0.0)
River herring, *Alosa aestivalis & pseudoharangus*	Fish	At‐risk	32 (23.5)	0 (0.0)
Southern Waternymph, *Najas guadalupensis*	Plant	Indicator	1 (0.7)	0 (0.0)
Spineless Hornwort, *Ceratophyllum echinatum*	Plant	Indicator	14 (10.3)	0 (0.0)
Striped Bass, *Morone saxatilis*	Fish	Indicator	6 (4.4)	0 (0.0)
Sturgeon, Genus: Acipenser	Fish	At‐risk	8 (5.9)	0 (0.0)

**TABLE 3:** Multi‐variable PERMANOVA (Adonis) analysis for Blue Catfish diets. We investigated differences between collection dates, life stage (juvenile or adult), size of fish (measured by 100 mm increments), and collection site. The single variable of collection date contributed the most significantly to variation in fish diet. *R* ^2^ scores indicate that collection date in conjunction with collection site further contributes to differences in diet. COI sequences suggest that life stage and size of fish also contribute to diet variation

Formula	18S		COI	
	*R* ^2^	*p*	*R* ^2^	*p*
CollectionDate	0.100	0.001	0.094	0.001
LifeStage	0.013	0.234	0.037	0.001
SizeRange	0.055	0.318	0.061	0.028
CollectionSite	0.015	0.139	0.013	0.109
CollectionDate:LifeStage	0.038	0.183	0.027	0.208
CollectionDate:SizeRange	0.119	0.182	0.102	0.038
CollectionDate:CollectionSite	0.045	0.013	0.039	0.002
LifeStage:CollectionSite	0.003	0.953	0.004	0.847
SizeRange:CollectionSite	0.029	0.489	0.019	0.681
CollectionDate:SizeRange:CollectionSite	0.048	0.767	0.033	0.934
Residuals	0.535	NA	0.572	NA
Total	1.000	NA	1.000	NA

**FIGURE 3:**
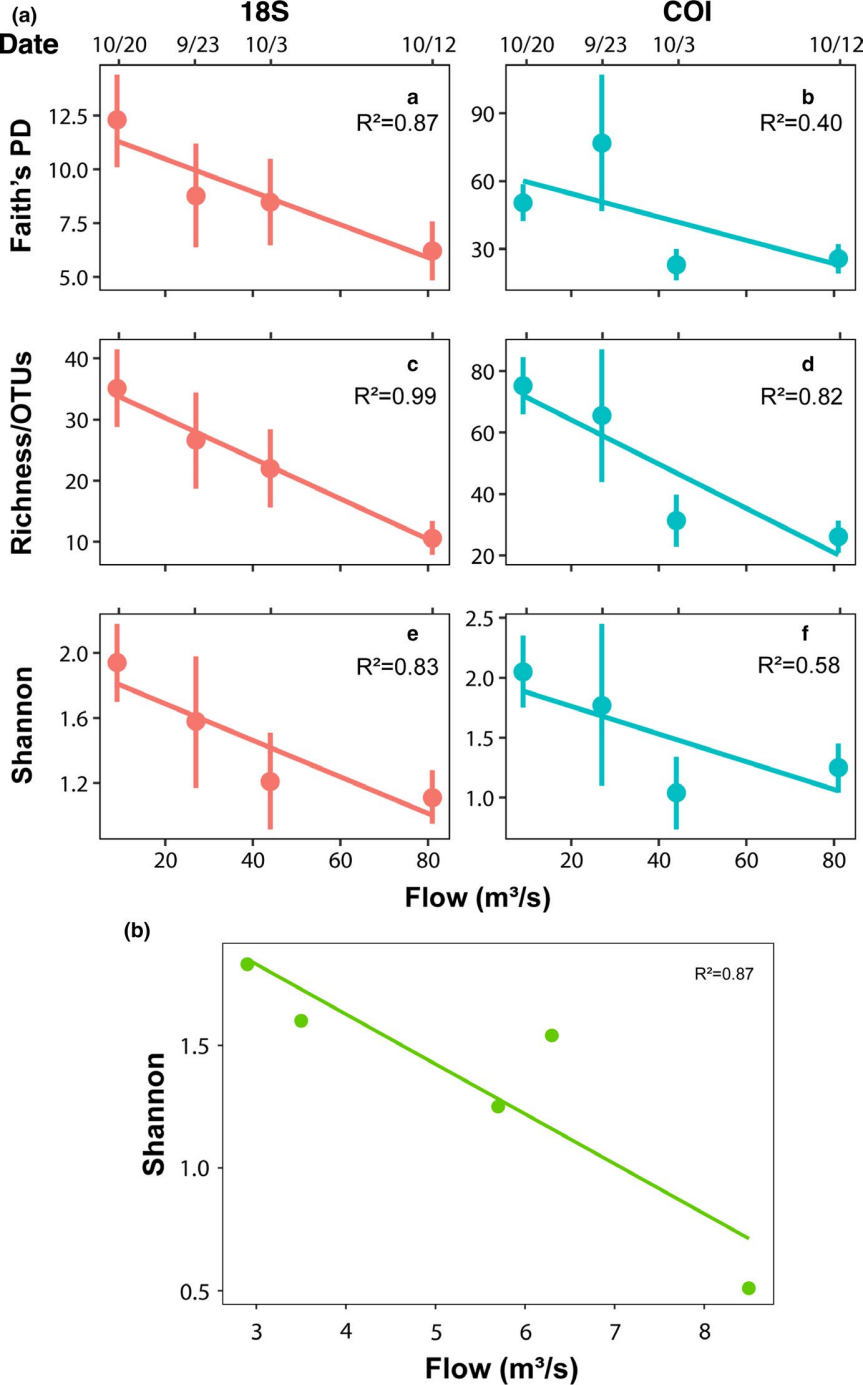
(a) Correlation between Blue Catfish diet richness and flow rates using high‐throughput sequencing. The relationship between flow rates and diet richness was calculated for Faith's phylogenetic diversity, richness as measured by observed operational taxonomic units (OTUs), and Shannon diversity index using both 18S (red) and COI (blue). All analyses demonstrate an inverse relationship where low flow rates are associated with increased diet diversity. (b) Correlation between Blue Catfish diet richness and flow rates using hybrid methodology. The relationship between flow rates and diet richness was calculated using the Shannon diversity index. Despite the lower richness detected with hybrid methods and the lower flow rates observed on sampling dates for this cohort, we observed the same inverse relationship where low flow rates are associated with increased diet diversity as seen with high‐throughput sequencing data

As collection date consistently appeared to be an important factor for Blue Catfish diet, we further investigated this metric. We noted that dates showing higher diversity (September 23 and October 20) had relatively low flow rates of 9.1 and 27.0 m^3^/s while October 3 and October 12 measured 81.3 and 44.2 m^3^/s, respectively. We compared the mean unique taxa identified at each date to the mean flow rate. Our results revealed a negative correlation between flow rate and Shannon diversity, richness, and PD (Figure 3a). ANCOM analysis indicated that algae and incidental diet items such as rotifers and ostracods were differentially abundant in the diets of fish collected during low flow rates.

### 3.3 Diet items of interest

Several indicator species for the York River Drainage and the Chesapeake Bay were identified in our taxonomic analysis including Striped Bass *Morone saxatilis*, Bay Anchovy *Anchoa mitchilli*, Blue Crab *Callinectes sapidus,* and native submersed aquatic vegetation (SAV; Table 2). We also identified consumption of invasive species such as Asian Clam *Corbicula Fluminea*, Red Swamp Crayfish *Procambaurs clarkii*, and *Hydrilla verticulata*. Lastly, we detected consumption of federally endangered Atlantic Sturgeon *Acipenser oxyrhynchus oxyrhynchus*, as well as at‐risk species such as river herring (Alewife *Alosa pseudoharengus* and Blueback Herring *Alosa aestivalis*) and American Shad *Alosa sapidissima*, species of concern under a moratorium in this geographical region due to low population levels (Greene et al., 2009).

### 3.4 Comparison of HTS and hybrid approach

**FIGURE 4:**
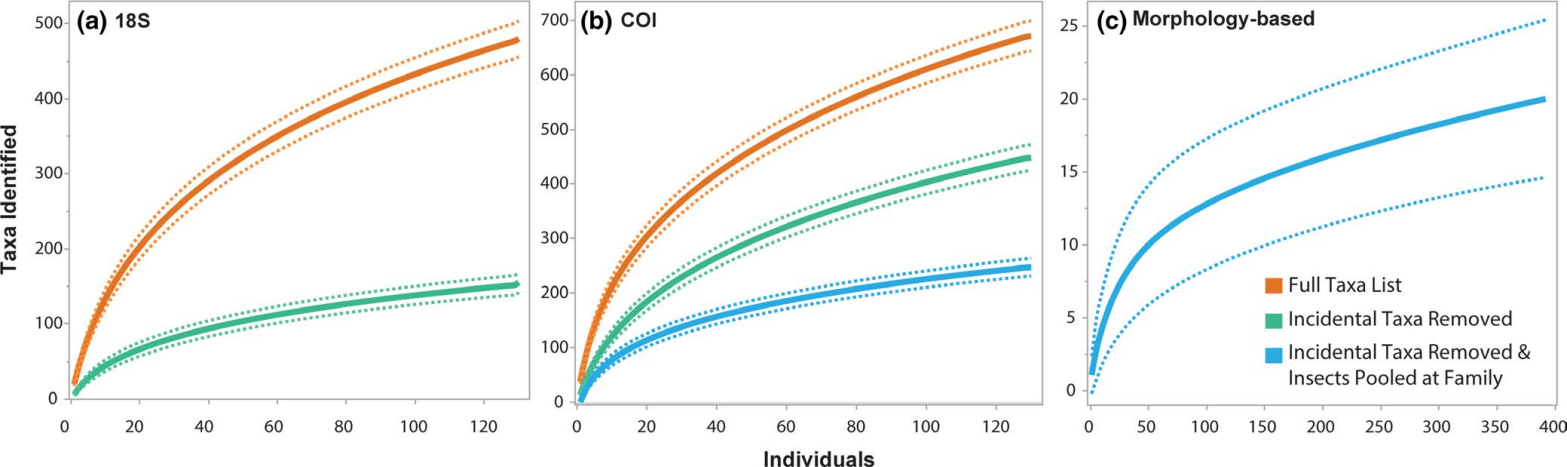
Cumulative prey curves. Cumulative prey curves were constructed for 18S, COI, and the hybrid morphology‐based method. As Blue Catfish do not intentionally prey upon incidental taxa and would therefore not have been counted in traditional morphological studies, they were removed from high‐throughput sequencing (HTS) data for direct comparison to the hybrid method. Additionally, insects were pooled to family level to match the taxonomic resolution used in the hybrid study. The resulting curve for 18S reached asymptote (B ≤ 0.05) at *N* = 67, COI at *N* = 114, and hybrid at *N* = 204. Both 18S and COI methods identified more unique taxa than the morphology study, yet reached asymptote with fewer sample numbers required, demonstrating that HTS outperforms traditional morphology‐based studies

We compared our taxonomic results to those achieved using a hybrid approach by Schmitt et al. (2018). The hybrid approach identified an average of 1.1 diet items per stomach. In contrast, HTS using DNA extracted from GI tracts resulted in an average of 45.3 COI OTUs and 22.7 18S OTUs. Taxonomic analysis indicated that the hybrid method failed to identify microscopic incidental diet items such as ciliates, apicomplexans, and rotifers as well as easily digestible taxa including fungi, sponges, and worms (Figure 2). Diets from the hybrid study skew heavily in favor of larger, slower to digest prey such as fish, crabs, and bivalves. Cumulative prey curves were developed for all methods (Figure 4). In order to accurately compare HTS to the hybrid dataset, we removed incidental taxa, including microscopic organisms and parasites, captured by HTS that would not have been the object of direct predation by Blue Catfish and therefore would not have been counted in a traditional morphological study. It was immediately apparent that cumulative prey curves are biased by the taxonomic resolution of the study; that is, tools (like HTS) that more precisely identify prey to species levels will inherently require more samples to achieve “sufficiency”. When this factor was accounted for by collapsing insects to family level instead of species level, HTS outperformed the hybrid approach by (a) identifying a greater number of prey taxa and (b) reaching an asymptote with fewer stomachs needed (*N* = 67 for 18S; *N* = 114 for COI, *N* = 214 for hybrid).

Lastly, we examined hybrid diet content in relation to flow rates as performed for HTS results. As seen with HTS data, samples collected on low flow rate days exhibited increased diet diversity when compared to samples collected on higher flow rate days (Figure 3b). Thus, the negative correlation between Blue Catfish diet diversity and flow rates was observed across multiple years using multiple methods.

## 4 DISCUSSION

Taxonomic analysis of diet contents using HTS revealed greater levels of at‐risk species consumption than observed using morphological or hybrid studies. The trophic ecology of invasive Blue Catfish has been investigated in several tidal rivers in Virginia (MacAvoy et al., 2001; Schloesser et al., 2011; Schmitt et al., 2019) and elsewhere in Chesapeake Bay (Aguilar et al., 2017; Iwanowicz et al., 2019) with high spatial and temporal resolution. Despite these efforts, federally endangered Atlantic Sturgeon predation was not detected through morphological identification or DNA barcoding of unidentified diet tissues. Similarly, samples used in the hybrid approach collected from the Pamunkey River did not detect predation of at‐risk American shad, Alewife, Blueback Herring, or American Eel *Anguilla rostrata*. However, HTS methodology detected considerable consumption of these species. Our study thus supports the hypothesis that traditional morphological and even hybrid approaches may miss important prey, especially small, soft‐bodied organisms, eggs, and larvae that digest rapidly (Bromley, 1994; Carreon‐Martinez et al., 2011; Legler et al., 2010; Schmitt et al., 2019). Future studies quantifying overall consumption of at‐risk species would be beneficial and would require tributary‐specific density calculations for both predator and prey species to determine impact levels.

Blue Catfish also predate heavily on indicator and invasive species within the Chesapeake Bay and its tidal tributaries. SAV was well‐represented, although primarily consisted of two species—native Spineless Hornwort *Ceratophyllum echinatum* and invasive Hydrilla. Invasive Asian Clam was the top diet item identified by genus in our study regardless of methodology. In contrast to the 81 identified instances of Asian Clam consumption, only 18 examples of mollusc prey other than Asian Clam were detected. The U.S. Fish and Wildlife Service has classified this species as high risk due to its ability to spread easily and outcompete native species (USFWS, 2015). Our study supports previous reports showing that Asian Clams are well‐established in the Chesapeake Bay watershed and may be outcompeting other mollusc species (Cerco & Noel, 2010; Freedman, 2013).

Our results suggest that Blue Catfish diet diversity is dependent on daily flow rates, with high flow rates resulting in lower diet diversity. This relationship held true across analysis methods, years, and flow magnitude. We hypothesize that Blue Catfish exhibit higher mobility and prey‐seeking behaviors during lower flow in the Pamunkey River. Conversely, they may decrease movement to conserve energy under higher flows, leading to lower probabilities of encountering diverse prey assemblages. Alternatively, higher flow rates could decrease consumption by interfering with Blue Catfish olfactory senses, by providing additional cover for prey through inundation of the aquatic‐terrestrial transition zone, or by washing off periphyton film from surfaces such as SAV, thereby decreasing incidental prey consumption.

Strong tidal influence occurs at our sampling sites and other tidal rivers in the Chesapeake Bay. The interplay between daily flow rate and tidal influence coupled with fine‐scale feeding ecology provides an opportunity for future research. For example, studies such as weekly feeding chronologies could be analyzed with HTS and correlated to tide schedule over a broad range of flow rates. Additional studies addressing gut evacuation rates in this species and ecosystem would also be of benefit to more precisely correlate flow rates, temperatures, and diet diversity.

Diet diversity analyses indicated multiple ontogenetic shifts as Blue Catfish size increased. Results indicate that juvenile diets are as diverse as those of adults, yet distinct with Asian Clam abundant in juvenile samples. Similarly, we noted no significant decrease in overall diversity as adult fish grew in length, but beta‐diversity analysis did indicate a shift toward piscivory around 500 mm TL. Schmitt et al. (2018) showed an ontogenetic diet shift from omnivory to piscivory between 500 and 900 mm TL depending on river, with Pamunkey River Blue Catfish shifting at 900 mm TL. It is important to note, however, that this shift was determined using *N* = 4,322 stomachs collected from tidal fresh, oligholaine, and mesohaline waters over the course of four field seasons, whereas the current study was completed at much finer spatiotemporal scales. However, the expanded ability to detect prey items coupled with the finer taxonomic resolution achieved through HTS may allow such ontogenetic shifts to be identified at earlier stages than previously determined. The limited spatiotemporal scope of the current study may also help explain why we observed ontogenetic shifts to piscivory at smaller sizes, as such shifts may be driven by seasonal and(or) spatial trends. Schmitt et al. (2018) defined piscivory as the probability that fish occurred in at least 50% of stomachs examined for a particular size range. However, all size ranges in our study, including our smallest of 100–200 mm TL, met this 50% criteria. These taxa detections may occur from consumption of eggs or larvae and would likely be missed using morphological techniques. As detection of prey life stage is not possible using HTS, these studies may need to use different parameters for defining piscivory, such as percent fish consumed in relation to total diet items.

The lack of decrease in diet diversity as Blue Catfish grew in size is surprising given that, as discussed above, Blue Catfish are generally understood to become less omnivorous and more piscivorous as they grow (Eggleton & Schramm, 2004; Graham, 1999; Schmitt et al., 2019). We may not have observed changes in diet diversity using HTS for several reasons. Firstly, our dataset only included 10 juvenile blue catfish. This small sample size may have made it impossible for us to detect changes in diet diversity between juveniles and adults. Our adult size classes were more evenly distributed, ranging from 14 samples to 38 samples per 100 mm TL grouping for COI, but would still benefit from additional sampling. Secondly, as previously noted, HTS will detect smaller prey that morphological studies could miss. Thus, Blue Catfish diets may remain more diverse across life stages than previously thought through the consumption of easily digestible prey and microscopic incidental diet items. Alternatively, diet diversity in larger fish using HTS methods may be inflated due to the inclusion of digested secondary prey items (diet items residing in the digestive track of the primary prey). For example, *Anguillicola crassus,* a top diet item identified in our Blue Catfish, is a parasitic nematode specific to American Eel (Warshafsky et al., 2019). Lastly, our fish length sample size is somewhat smaller than those used in other studies. Schmitt et al. (2018) found that fish in the Pamunkey River became mostly piscivorous at around 900 mm TL, whereas the largest Blue Catfish in our diet set was only 770 mm TL. It may be that true piscivory, and the concurrent expected decrease in diet diversity, had not yet been reached with our sample.

Despite the larger hybrid diet dataset, we found that HTS provided significantly higher diversity and richness estimates, and ultimately a broader and more holistic view of Blue Catfish diets. The number of taxa detected increased 20‐ to 40‐fold when using HTS on GI tracts in comparison with hybrid methodology examining stomachs alone. Given the large disparities in diet composition across approaches and the documented abilities of morphology‐based methods relative to DNA‐based studies, the differences found here are not likely due to annual variation in Blue Catfish diets. Similar observations have been made for other predator species and ecosystems previously examined (Cowart et al., 2015; Jakubavičiūtė et al., 2017) due to the inability of traditional morphological analyses to identify partially digested items, microscopic organisms, and rapidly digested tissue such as eggs and larvae (Guillerault et al., 2017; Su et al., 2018). Cumulative prey curves demonstrate that HTS requires approximately half the samples needed for morphology‐based studies to capture full diet diversity for Blue Catfish.

It is important to note that HTS diversity and richness calculations may be inflated due to the detection of incidental and secondary diet items. For example, SAV contains high biomass of species‐rich periphyton (biofilm) on leaf and stem surfaces that contain a diverse assemblage of algae, cyanobacteria, heterotrophic microbes, and detritus (Gordon‐Bradley et al., 2014; Hoagland et al., 1982). Thus, SAV provides habitat and food resources for macroinvertebrates, zooplankton, and small fishes. As omnivorous fishes feed on and among these SAV beds, they are also passively consuming incidental organisms within this periphyton assemblage that are likely contributing to the high diet diversity observed with HTS results in comparison with the hybrid approach. Furthermore, the use of GI tracts in this study, while increasing the ability to detect primary prey consumed over a longer time frame, allows for mixing of primary and secondary prey DNA as items are digested. However, Jakubavičiūtė et al. (2017) hypothesized that secondary prey contribution to diet diversity would be minimal given their decreased biomass and advanced DNA degradation. Further studies examining the contribution of incidental and secondary prey to predator diets would be of interest as the use of HTS to examine diet diversity increases.

While our study indicates that HTS methods are generally superior to morphological methods given the lower sample sizes required as well as the breadth of results achieved, there may be instances in which traditional analyses are still appropriate. For instance, many diet studies depend on data such as biomass and direct counts (Christensen, 2009; Hyslop, 1980). Additionally, Jakubavičiūtė et al. (2017) noted that morphological analysis identified some species missed by HTS. Likewise, a cursory morphological analysis of diet content in our study noted a few items not identified by HTS, the most common instances being molluscs, crayfish, and plants. These items were most likely missed due either to primer mismatch or to instances where tissue and DNA had degraded, but DNA‐deficient parts such as shells and exoskeletons remained. Lastly, instances of Blue Catfish cannibalism (Jennings et al., 2018; Schmitt et al., 2018) may be detected morphometrically, but will be indistinguishable from host DNA using HTS. Therefore, the utility of traditional diet studies remains depending on objective. Cowart et al. (2015) suggested “double inventories” (morphological and molecular) would provide reference data for future “blind metabarcode” surveys.

Researchers wishing to conduct HTS diet studies should consider taxonomic targets and desired resolution early in planning stages (Alberdi et al., 2019). Our study employed two genes traditionally used for barcoding species, mitochondrial cytochrome c oxidase I (COI) and the 18S ribosomal RNA gene. In contrast to results published by Iwanowicz et al. (2019), a parallel comparison of COI and 18S sequences for Blue Catfish diet demonstrated the vastly different conclusions that may be drawn from a diet study if only one gene is utilized. An examination at the phylum level using COI would indicate that the majority of Blue Catfish diet consists of arthropods, with significant contributions from chordates, and molluscs. The 18S analysis revealed a more complicated and diverse diet, with many more phyla evenly contributing to Blue Catfish diet, including streptophyta, nematoda, and chlorophyta, none of which were indicated to a significant extent when using COI. Additionally, analysis at the genus level revealed that, other than Asian Clam, completely distinct top prey items were identified with each of our three methods (18S, COI, or hybrid). Thus, target species should be carefully considered when choosing a barcoding gene, and we recommend more than one gene be used to capture full diet diversity.

In addition to preferential amplification of species, the two genes used in this study exhibited differences in taxonomic resolution of diet items. As seen in previous studies (Günther et al., 2018; Holman et al., 2019), the more conserved 18S rRNA gene amplified across a broader range of taxa, leading to greater diversity values when examined at high taxonomic levels in comparison with COI. The broader amplification range of 18S also allowed for higher detection of incidental diet items, which researchers may or may not want to include depending on study objectives. Conversely, the increased sequence variation of COI allowed for a more finite resolution of successfully amplified taxa and ultimately led to a higher number of detected species, despite its narrower phylogenetic scope. The fine‐scale taxonomic resolution achieved with COI also detected differences in alpha and beta diversities that were not detected using the more conserved 18S gene.

Our dataset contained many COI sequences that could not be taxonomically assigned to a phylum. These unassigned COI sequences reflect the degree to which the wealth of biodiversity has yet to be genetically catalogued. Mora et al. (2011) hypothesized that only 13.8% of species on earth have been described. Another study estimated that in freshwater systems, approximately 3,000 fish and 100 bivalves remain undescribed (Tedesco et al., 2014). With the advent of metabarcoding, we now have the ability to genetically detect these undescribed creatures. Moving forward, it will be important to curate vouchered specimens with DNA sequences as we seek to increase our knowledge of living organisms and predator–prey interactions.

## CONFLICT OF INTEREST

The authors have no conflicts of interest to declare.

## AUTHOR CONTRIBUTION

**Heather Evans:** Data curation (lead); Formal analysis (lead); Investigation (lead); Methodology (lead); Project administration (lead); Software (equal); Supervision (equal); Writing‐original draft (equal); Writing‐review & editing (lead). **Aaron Bunch:** Conceptualization (lead); Formal analysis (equal); Funding acquisition (lead); Project administration (equal); Supervision (equal); Writing‐original draft (equal); Writing‐review & editing (supporting). **Joseph Schmitt:** Formal analysis (equal); Funding acquisition (equal); Investigation (equal); Methodology (equal); Writing‐original draft (equal); Writing‐review & editing (equal). **Frederick Hoogakker:** Data curation (supporting); Investigation (supporting); Writing‐review & editing (supporting). **Kara Carlson:** Data curation (equal); Investigation (equal); Methodology (supporting); Writing‐review & editing (supporting).

## Data Availability

Data available from OSF: https://osf.io/g6wcq. Evans, H., & Bunch, A. High throughput dual gene sequencing of Pamunkey River (Virginia, USA) tidal‐fresh fish diets.

## References

[ece37460-bib-0001] Aguilar, R. , Ogburn, M. B. , Driskell, A. C. , Weigt, L. A. , Groves, M. C. , & Hines, A. H. (2017). Gutsy genetics: Identification of digested piscine prey items in the stomach contents of sympatric native and introduced warmwater catfishes via DNA barcoding. Environmental Biology of Fishes, 100(4), 325–336. 10.1007/s10641-016-0523-8

[ece37460-bib-0002] Alberdi, A. , Aizpurua, O. , Bohmann, K. , Gopalakrishnan, S. , Lynggaard, C. , Nielsen, M. , & Gilbert, M. T. P. (2019). Promises and pitfalls of using high‐throughput sequencing for diet analysis. Molecular Ecology Resources, 19(2), 327–348. 10.1111/1755-0998.12960 30358108

[ece37460-bib-0003] Benjamini, Y. , & Hochberg, Y. (1995). Controlling the false discovery rate: A practical and powerful approach to multiple testing. Journal of the Royal Statistical Society: Series B (Methodological), 57(1), 289–300. 10.1111/j.2517-6161.1995.tb02031.x

[ece37460-bib-0004] Bessey, C. , Jarman, S. N. , Stat, M. , Rohner, C. A. , Bunce, M. , Koziol, A. , & Berry, O. (2019). DNA metabarcoding assays reveal a diverse prey assemblage for Mobula rays in the Bohol Sea, Philippines. Ecology and Evolution, 9(5), 2459–2474. 10.1002/ece3.4858 30891193 PMC6405500

[ece37460-bib-0005] Bizzarro, J. J. , Robinson, H. J. , Rinewalt, C. S. , & Ebert, D. A. (2007). Comparative feeding ecology of four sympatric skate species off central California, USA. Environmental Biology of Fishes, 80(2–3), 197–220. 10.1007/s10641-007-9241-6

[ece37460-bib-0006] Bolyen, E. , Rideout, J. R. , Dillon, M. R. , Bokulich, N. A. , Abnet, C. , Al‐Ghalith, G. A. , & Caporaso, J. G. (2019). Reproducible, interactive, scalable, and extensible microbiome data science using QIIME 2. Nature Biotechnology, 37, 852–857. 10.7287/peerj.preprints.27295v1 PMC7015180 31341288

[ece37460-bib-0007] Bromley, P. J. (1994). The role of gastric evacuation experiments in quantifying the feeding rates of predatory fish. Reviews in Fish Biology and Fisheries, 4(1), 36–66. 10.1007/BF00043260

[ece37460-bib-0008] Brown, S. C. , Bizzarro, J. J. , Cailliet, G. M. , & Ebert, D. A. (2012). Breaking with tradition: Redefining measures for diet description with a case study of the Aleutian skate *Bathyraja* *aleutica* (Gilbert 1896). Environmental Biology of Fishes, 95(1), 3–20. 10.1007/s10641-011-9959-z

[ece37460-bib-0010] Bunch, A. J. , Greenlee, R. S. , & Brittle, E. M. (2018). Blue catfish density and biomass in a tidal tributary in coastal Virginia. Northeastern Naturalist, 25(2), 333–340. 10.1656/045.025.0215

[ece37460-bib-0011] Callahan, B. J. , McMurdie, P. J. , Rosen, M. J. , Han, A. W. , Johnson, A. J. A. , & Holmes, S. P. (2016). DADA2: High‐resolution sample inference from Illumina amplicon data. Nature Methods, 13(7), 581. 10.1038/nmeth.3869 27214047 PMC4927377

[ece37460-bib-0012] Camacho, C. , Coulouris, G. , Avagyan, V. , Ma, N. , Papadopoulos, J. , Bealer, K. , & Madden, T. L. (2009). BLAST+: Architecture and applications. BMC Bioinformatics, 10, 421. 10.1186/1471-2105-10-421 20003500 PMC2803857

[ece37460-bib-0013] Carreon‐Martinez, L. , Johnson, T. B. , Ludsin, S. A. , & Heath, D. D. (2011). Utilization of stomach content DNA to determine diet diversity in piscivorous fishes. Journal of Fish Biology, 78(4), 1170–1182. 10.1111/j.1095-8649.2011.02925.x 21463313

[ece37460-bib-0014] Casey, J. M. , Meyer, C. P. , Morat, F. , Brandl, S. J. , Planes, S. , & Parravicini, V. (2019). Reconstructing hyperdiverse food webs: Gut content metabarcoding as a tool to disentangle trophic interactions on coral reefs. Methods in Ecology and Evolution, 10(8), 1157–1170. 10.1111/2041-210X.13206

[ece37460-bib-0015] Cerco, C. F. , & Noel, M. R. (2010). Monitoring, modeling, and management impacts of bivalve filter feeders in the oligohaline and tidal fresh regions of the Chesapeake Bay system. Ecological Modelling, 221(7), 1054–1064. 10.1016/j.ecolmodel.2009.07.024

[ece37460-bib-0016] Chesapeake Bay Program (2020). Invasive Catfish Management Strategy. Retrieved from https://www.chesapeakebay.net/documents/Invasive_Catfish_Management_Strategy_Aug_2020_final.pdf

[ece37460-bib-0017] Christensen, V. (2009). Fisheries ecosystem model of the Chesapeake Bay. National Oceanic and Atmospheric Administration, (October). Retrieved from http://books.google.com/books?id=_M3LYgEACAAJ&dq=intitle:fisheries+ecosystem+model+of+the+inauthor:christensen&hl=&cd=1&source=gbs_api

[ece37460-bib-0018] Colwell, R. K. (2013). EstimateS: Statistical estimation of species richness and shared species from samples. Retrieved from http://purl.oclc.org/estimates

[ece37460-bib-0019] Cowart, D. A. , Pinheiro, M. , Mouchel, O. , Maguer, M. , Grall, J. , Miné, J. , & Arnaud‐Haond, S. (2015). Metabarcoding is powerful yet still blind: A comparative analysis of morphological and molecular surveys of seagrass communities. PLoS One, 10(2), 1–26. 10.1371/journal.pone.0117562 PMC4323199 25668035

[ece37460-bib-0020] Eggleton, M. A. , & Schramm, H. L. (2004). Feeding ecology and energetic relationships with habitat of blue catfish, *Ictalurus* *furcatus*, and flathead catfish, *Pylodictis* *olivaris*, in the lower Mississippi River, U.S.A. Environmental Biology of Fishes, 70(2), 107–121. 10.1023/B:EBFI.0000029341.45030.94

[ece37460-bib-0021] Fabrizio, M. C. , Tuckey, T. D. , Latour, R. J. , White, G. C. , & Norris, A. J. (2018). Tidal habitats support large numbers of invasive Blue Catfish in a Chesapeake Bay Subestuary. Estuaries and Coasts, 41(3), 827–840. 10.1007/s12237-017-0307-1

[ece37460-bib-0022] Freedman, M. R. (2013). Distribution and Impacts of Invasive Bivalve *Corbicula* *fluminea* in Tidal Freshwater York River Tributaries. *Dissertations, Theses, and Masters Projects, Paper 1539*. 10.25773/v5-vt43-9x26

[ece37460-bib-0023] Gordon‐Bradley, N. , Lymperopoulou, D. S. , & Williams, H. N. (2014). Differences in bacterial community structure on *Hydrilla* *verticillata* and *Vallisneria* *americana* in a freshwater spring. Microbes and Environments, 29(1), 67–73. 10.1264/jsme2.ME13064 24553106 PMC4041241

[ece37460-bib-0024] Graham, K. (1999). A review of the biology and management of Blue Catfish. American Fisheries Society Symposium, 24, 37–49.

[ece37460-bib-0025] Greene, K. E. , Zimmerman, J. L. , Laney, R. W. , & Thomas‐Blate, J. C. (2009). Atlantic coast diadromous fish habitat: A review of utilization, threats, recommendations for conservation, and research needs. Atlantic States Marine Fisheries Commission, Series #9.

[ece37460-bib-0026] Guillerault, N. , Bouletreau, S. , Iribar, A. , Valentini, A. , & Santoul, F. (2017). Application of DNA metabarcoding on faeces to identify European catfish *Silurus* *glanis* diet. Journal of Fish Biology, 90(5), 2214–2219. 10.1111/jfb.13294 28345142

[ece37460-bib-0027] Günther, B. , Knebelsberger, T. , Neumann, H. , Laakmann, S. , & Martínez Arbizu, P. (2018). Metabarcoding of marine environmental DNA based on mitochondrial and nuclear genes. Scientific Reports, 8(1), 1–13. 10.1038/s41598-018-32917-x 30287908 PMC6172225

[ece37460-bib-0028] Harms‐Tuohy, C. A. , Schizas, N. V. , & Appeldoorn, R. S. (2016). Use of DNA metabarcoding for stomach content analysis in the invasive lionfish *Pterois* *volitans* in Puerto Rico. Marine Ecology Progress Series, 558, 181–191. 10.3354/meps11738

[ece37460-bib-0029] Hoagland, K. D. , Roemer, S. C. , & Rosowski, J. R. (1982). Colonization and community structure of two periphyton assemblages, with emphasis on the diatoms (Bacillariophyceae). American Journal of Botany, 69(2), 188. 10.2307/2443006

[ece37460-bib-0030] Holman, L. E. , De Bruyn, M. , Creer, S. , Carvalho, G. , Robidart, J. , & Rius, M. (2019). Detection of introduced and resident marine species using environmental DNA metabarcoding of sediment and water. Scientific Reports, 9(1), 11559. 10.1038/s41598-019-47899-7 31399606 PMC6689084

[ece37460-bib-0031] Hyslop, E. J. (1980). Stomach contents analysis—A review of methods and their application. Journal of Fish Biology, 17(4), 411–429. 10.1111/j.1095-8649.1980.tb02775.x

[ece37460-bib-0032] Iwanowicz, D. D. , Schill, W. B. , Sanders, L. R. , Groves, T. , & Groves, M. C. (2019). Establishing molecular methods to quantitatively profile gastric diet items of fish—Application to the invasive blue catfish (*Ictalurus**furcatus*). U.S. Geological Survey Open‐File Report. 10.3133/ofr20191021

[ece37460-bib-0033] Jakubavičiūtė, E. , Bergström, U. , Eklöf, J. S. , Haenel, Q. , & Bourlat, S. J. (2017). DNA metabarcoding reveals diverse diet of the three‐spined stickleback in a coastal ecosystem. PLoS One, 12(10), e0186929. 10.1371/journal.pone.0186929 29059215 PMC5653352

[ece37460-bib-0034] Jennings, C. A. , Mitchell, G. E. , & Nelson, C. (2018). Seasonal food habits of introduced blue catfish in Lake Oconee, Georgia. JSAFWA: Journal of the Southeastern Association of Fish and Wildlife Agencies, 5, 39–45.

[ece37460-bib-0035] Legler, N. D. , Johnson, T. B. , Heath, D. D. , & Ludsin, S. A. (2010). Water temperature and prey size effects on the rate of digestion of larval and early juvenile fish. Transactions of the American Fisheries Society, 139(3), 868–875. 10.1577/t09-212.1

[ece37460-bib-0036] Leray, M. , Agudelo, N. , Mills, S. C. , & Meyer, C. P. (2013). Effectiveness of annealing blocking primers versus restriction enzymes for characterization of generalist diets: Unexpected prey revealed in the gut contents of two coral reef fish species. PLoS One, 8(4), e58076. 10.1371/journal.pone.0058076 23579925 PMC3620324

[ece37460-bib-0037] Leray, M. , & Knowlton, N. (2015). DNA barcoding and metabarcoding of standardized samples reveal patterns of marine benthic diversity. Proceedings of the National Academy of Sciences, 2014, 201424997. 10.1073/pnas.1424997112 PMC4343139 25646458

[ece37460-bib-0038] MacAvoy, S. E. , Macko, S. A. , & Garman, G. C. (2001). Isotopic turnover in aquatic predators: Quantifying the exploitation of migratory prey. Canadian Journal of Fisheries and Aquatic Sciences, 58(5), 923–932. 10.1139/f01-045

[ece37460-bib-0039] Mandal, S. , Van Treuren, W. , White, R. A. , Eggesbø, M. , Knight, R. , & Peddada, S. D. (2015). Analysis of composition of microbiomes: A novel method for studying microbial composition. Microbial Ecology in Health & Disease, 26, 1–7. 10.3402/mehd.v26.27663 PMC4450248 26028277

[ece37460-bib-0040] Mora, C. , Tittensor, D. P. , Adl, S. , Simpson, A. G. B. , & Worm, B. (2011). How many species are there in earth and in the ocean? PLoS Biology, 9(8), e1001127. 10.1371/journal.pbio.1001127 21886479 PMC3160336

[ece37460-bib-0041] Moran, Z. , Orth, D. J. , Schmitt, J. D. , Hallerman, E. M. , & Aguilar, R. (2015). Effectiveness of DNA barcoding for identifying piscine prey items in stomach contents of piscivorous catfishes. Environmental Biology of Fishes, 99(1), 161–167. 10.1007/s10641-015-0448-7

[ece37460-bib-0042] Quast, C. , Pruesse, E. , Yilmaz, P. , Gerken, J. , Schweer, T. , Yarza, P. , & Glöckner, F. O. (2013). The SILVA ribosomal RNA gene database project: Improved data processing and web‐based tools. Nucleic Acids Research, 41(Database issue), D590–D596. 10.1093/nar/gks1219 23193283 PMC3531112

[ece37460-bib-0043] Rees, G. N. , Shackleton, M. E. , Watson, G. O. , Dwyer, G. K. , & Stoffels, R. J. (2020). Metabarcoding demonstrates dietary niche partitioning in two coexisting blackfish species. Marine and Freshwater Research, 71(4), 512. 10.1071/MF18491

[ece37460-bib-0044] Rognes, T. , Flouri, T. , Nichols, B. , Quince, C. , & Mahé, F. (2016). VSEARCH: A versatile open source tool for metagenomics. PeerJ, 4, e2584. 10.7717/peerj.2584 27781170 PMC5075697

[ece37460-bib-0045] Schloesser, R. W. , Fabrizio, M. C. , Latour, R. J. , Garman, G. C. , Greenlee, B. , Groves, M. , & Gartland, J. (2011). Ecological role of blue catfish in Chesapeake Bay communities and implications for management. American Fisheries Society Symposium, 77, 1–14.

[ece37460-bib-0046] Schmitt, J. D. , Hallerman, E. M. , Bunch, A. , Moran, Z. , Emmel, J. A. , & Orth, D. J. (2017). Predation and prey selectivity by nonnative catfish on migrating alosines in an Atlantic slope estuary. Marine and Coastal Fisheries, 9(1), 108–125. 10.1080/19425120.2016.1271844

[ece37460-bib-0047] Schmitt, J. D. , Peoples, B. K. , Bunch, A. J. , Castello, L. , & Orth, D. J. (2019). Modeling the predation dynamics of invasive blue catfish (*Ictalurus* *furcatus*) in Chesapeake Bay. Fishery Bulletin, 117(4), 277–290. 10.7755/FB.117.4.1

[ece37460-bib-0048] Schmitt, J. D. , Peoples, B. K. , Castello, L. , & Orth, D. J. (2018). Feeding ecology of generalist consumers: A case study of invasive blue catfish *Ictalurus* *furcatus* in Chesapeake Bay, Virginia, USA. Environmental Biology of Fishes, 102(3), 443–465. 10.1007/s10641-018-0783-6

[ece37460-bib-0049] Schultz, A. A. , Kumagai, K. K. , & Bridges, B. B. (2015). Methods to evaluate gut evacuation rates and predation using acoustic telemetry in the Tracy Fish Collection Facility primary channel. Animal Biotelemetry, 3, 13. 10.1186/s40317-015-0034-y

[ece37460-bib-0050] Schwarz, D. , Spitzer, S. M. , Thomas, A. C. , Kohnert, C. M. , Keates, T. R. , & Acevedo‐Gutiérrez, A. (2018). Large‐scale molecular diet analysis in a generalist marine mammal reveals male preference for prey of conservation concern. Ecology and Evolution, 8(19), 9889–9905. 10.1002/ece3.4474 30386584 PMC6202700

[ece37460-bib-0051] Sousa, L. L. , Xavier, R. , Costa, V. , Humphries, N. E. , Trueman, C. , Rosa, R. , & Queiroz, N. (2016). DNA barcoding identifies a cosmopolitan diet in the ocean sunfish. Scientific Reports, 6, 1–9. 10.1038/srep28762 27373803 PMC4931451

[ece37460-bib-0052] Su, M. , Liu, H. , Liang, X. , Gui, L. , & Zhang, J. (2018). Dietary analysis of marine fish species: Enhancing the detection of prey‐specific DNA sequences via high‐throughput sequencing using blocking primers. Estuaries and Coasts, 41(2), 560–571. 10.1007/s12237-017-0279-1

[ece37460-bib-0053] Tedesco, P. A. , Bigorne, R. , Bogan, A. E. , Giam, X. , Jézéquel, C. , & Hugueny, B. (2014). Estimating how many undescribed species have gone extinct. Conservation Biology, 28(5), 1360–1370. 10.1111/cobi.12285 24684650

[ece37460-bib-0054] Tverin, M. , Esparza‐Salas, R. , Strömberg, A. , Tang, P. , Kokkonen, I. , Herrero, A. , & Lundström, K. (2019). Complementary methods assessing short and long‐term prey of a marine top predator – Application to the grey seal‐fishery conflict in the Baltic Sea. PLoS One, 14(1), e0208694. 10.1371/journal.pone.0208694 30601857 PMC6314633

[ece37460-bib-0055] USFWS (2015). Asian Clam (*Corbicula**fluminea*) 1 Native Range, and Status in the United States Native Range.

[ece37460-bib-0056] Waraniak, J. M. , Baker, E. A. , & Scribner, K. T. (2018). Molecular diet analysis reveals predator–prey community dynamics and environmental factors affecting predation of larval lake sturgeon *Acipenser* *fulvescens* in a natural system. Journal of Fish Biology, 93(4), 616–629. 10.1111/jfb.13726 29956319

[ece37460-bib-0057] Waraniak, J. M. , Marsh, T. L. , & Scribner, K. T. (2019). 18S rRNA metabarcoding diet analysis of a predatory fish community across seasonal changes in prey availability. Ecology and Evolution, 9(3), 1410–1430. 10.1002/ece3.4857 30805170 PMC6374664

[ece37460-bib-0058] Warshafsky, Z. T. , Tuckey, T. D. , Vogelbein, W. K. , Latour, R. J. , & Wargo, A. R. (2019). Temporal, spatial, and biological variation of nematode epidemiology in American eels. Canadian Journal of Fisheries and Aquatic Sciences, 76(10), 1808–1818. 10.1139/cjfas-2018-0136

[ece37460-bib-0059] Waters, D. S. , Kwak, T. J. , Arnott, J. B. , & Pine, W. E. (2004). Evaluation of stomach tubes and gastric lavage for sampling diets from blue catfish and flathead catfish. North American Journal of Fisheries Management, 24(1), 258–261. 10.1577/m02-156

[ece37460-bib-0060] Wickham, H. (2016). Introduction. ggplot2: Elegant graphics for data analysis. Springer‐Verlag New York.

[ece37460-bib-0061] Zhan, A. , Bailey, S. A. , Heath, D. D. , & Macisaac, H. J. (2014). Performance comparison of genetic markers for high‐throughput sequencing‐based biodiversity assessment in complex communities. Molecular Ecology Resources, 14, 1049–1059. 10.1111/1755-0998.12254 24655333

